# Crystal structure of a nickel compound comprising two nickel(II) complexes with different ligand environments: [Ni(tren)(H_2_O)_2_][Ni(H_2_O)_6_](SO_4_)_2_

**DOI:** 10.1107/S2056989020001358

**Published:** 2020-02-06

**Authors:** Karilys González Nieves, Dalice M. Piñero Cruz

**Affiliations:** a Department of Natural Sciences, University of Puerto Rico, Carolina Campus, 2100 Avenida Sur, Carolina, PR 00987, Puerto Rico; b Department of Chemistry, University of Puerto Rico, Rio Piedras Campus, Ponce de Leon Avenue, San Juan, PR 00931, Puerto Rico

**Keywords:** crystal structure, nickel complexes, tren, tripodal ligand, hydrogen bonding

## Abstract

The title compound, di­aqua­[tris­(2-amino­eth­yl)amine]­nickel(II) hexa­aqua­nickel(II) bis­(sulfate), [Ni(C_6_H_18_N_4_)(H_2_O)_2_][Ni(H_2_O)_6_](SO_4_)_2_ or [Ni(tren)(H_2_O)_2_][Ni(H_2_O)_6_](SO_4_)_2_, consists of two octa­hedral nickel complexes within the same unit cell. These metal complexes are formed from the reaction of [Ni(H_2_O)_6_](SO_4_) and the ligand tris­(2-amino­eth­yl)amine (tren). The crystals of the title compound are purple, different from those of the starting complex [Ni(H_2_O)_6_](SO_4_), which are turquoise. The reaction was performed both in a 1:1 and 1:2 metal–ligand molar ratio, always yielding the co-precipitation of the two types of crystals. The asymmetric unit of the title compound, which crystallizes in the space group *Pnma*, consists of two half Ni^II^ complexes and a sulfate counter-anion. The mononuclear cationic complex [Ni(tren)(H_2_O)_2_]^2+^ comprises an Ni ion, the tren ligand and two water mol­ecules, while the mononuclear complex [Ni(H_2_O)_6_]^2+^ consists of another Ni ion surrounded by six coordinated water mol­ecules. The [Ni(tren)(H_2_O)_2_] and [Ni(H_2_O)_6_] subunits are connected to the SO_4_^2−^ counter-anions through hydrogen bonding, thus consolidating the crystal structure.

## Abstract 2

The title compound, [Ni(tren)(H_2_O)_2_][Ni(H_2_O)_6_](SO_4_)_2_, consists of two octa­hedral nickel complexes within the same unit cell. It co-precipitates with the starting material, [Ni(H_2_O)_6_](SO_4_). The crystals of the title compound are purple, different from the [Ni(H_2_O)_6_](SO_4_) crystals, which are turquoise.

## Chemical context   

Tris(2-amino­eth­yl)amine (tren) has been used extensively as an ancillary tripodal ligand for capping transition metals to form mononuclear and polynuclear complexes. The tren ligand has the capacity to chelate metal ions through its central tertiary amine and through its three terminal amine groups in a spider-like conformation, leaving one or two positions available for additional ligand coordination (Marzotto *et al.*, 1993 ▸; Albertin *et al.*, 1975 ▸; Blackman, 2005 ▸; Brines *et al.*, 2007 ▸). Metal complexes with a variety of ligands in which also tren is coordinating to the metal center have been proposed for applications in catalysis (Ruffin *et al.*, 2017 ▸), sensors, and as precursors of bioinorganic reactions (Sakai *et al.*, 1996 ▸). For instance, Ni(tren) complexes have been proposed for applications in biological systems (Salam & Aoki, 2001 ▸) or as a model to study enanti­oselective synthesis or asymmetric catalysis (Rao *et al.*, 2009 ▸), and as coordination polymers in magnetism, electrical conductivity and ion exchange (Park *et al.*, 2001 ▸; Tanase *et al.*, 1996 ▸). [Ni(tren)(H_2_O)_2_] was reported previously (Chen *et al.*, 2001 ▸; Pedersen *et al.*, 2014 ▸); however, to our knowledge, this is the first report of it co-crystallizing with the hexa­aquo nickel complex [Ni(H_2_O)_6_](SO_4_).
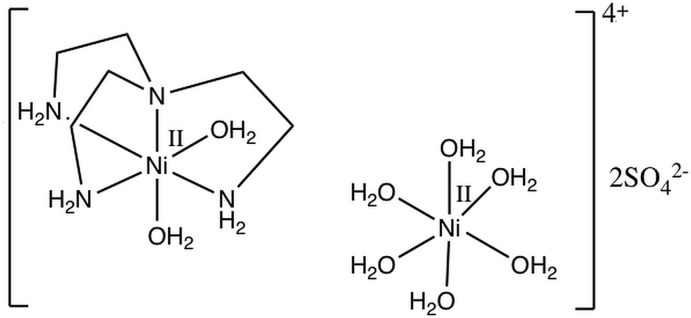


## Structural commentary   

**Figure 1:**
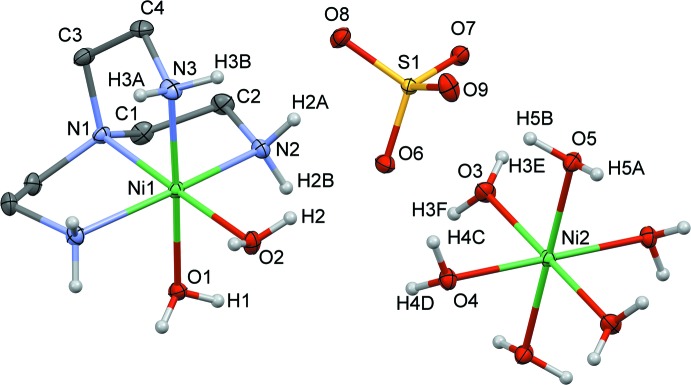
View of the mol­ecular structure of the title compound with displacement ellipsoids drawn at the 20% probability level and labeling scheme for the symmetry-independent atoms. The CH_2_ hydrogen atoms have been omitted for clarity. The symmetry operations generating the equivalent atoms are 1 − *x*, 1 − *y*, 2 − *z* and *x*, 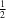 − *y*, *z* for [Ni(H_2_O)_6_]^2+^ and [Ni(tren)(H_2_O)_2_]^2+^, respectively.

Fig. 1 ▸ shows the molecular structure of the title compound, which crystallizes in the space group *Pnma*. Its asymmetric unit comprises two half Ni^II^ complexes and a sulfate counter-anion. Each Ni complex shows a different ligand environment: (i) the mononuclear cationic complex [Ni(tren)(H_2_O)_2_]^2+^ includes Ni1, the tren ligand and two water mol­ecules; (ii) the mononuclear complex [Ni(H_2_O)_6_]^2+^ consists of Ni2 surrounded by six coordinated water mol­ecules.

Ni1 exhibits an octa­hedral geometry of the type N_4_O_2_, with the central N1 atom of the tren ligand occupying one of the axial positions and atoms N2, N3 and N2^i^ occupying three of the equatorial positions [symmetry code: (i) *x*, −*y* + 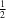, *z*]. The remaining two positions, one axial (O2) and one equatorial (O1), are occupied by two oxygen atoms from the two water mol­ecules. The bond lengths are similar for the Ni1—N bonds that are *trans* to oxygen atoms; for instance, Ni1—N1_ax_ is 2.064 (2) Å and Ni1—N3_eq_ is 2.069 (2) Å; a longer bond distance is observed between Ni1—N2_eq_, 2.122 (2), which is *trans* by symmetry to another nitro­gen atom, N2^i^. The nickel–oxygen bond length is shorter for Ni1—O2_ax_ at 2.094 (2) Å, in comparison to Ni1—O1_eq_, which is 2.140 (2) Å. The N3 and C3 atoms of the tren ligand lie on a mirror plane perpendicular to [010]. This results in a symmetry-induced disorder of the N3/C4/C3 fragment. The octa­hedral geometry around the Ni1 ion is reflected by the angles N1—Ni1—O2 = 178.42 (8)°, N2—Ni1—N2^i^ = 164.74 (9)°, and N3—Ni1—O1 = 177.27 (8)°.

The Ni2 ion of the mononuclear complex [Ni(H_2_O)_6_]^2+^ also shows an octa­hedral geometry. In the asymmetric unit, the atom Ni2 sits on an inversion center on a screw axis along the *b*-axis direction. The Ni2—O_water_ bond lengths with O3, O4 and O5 range between 2.051 (1) and 2.074 (1) Å, respectively, with angles of 180° due to symmetry.

## Supra­molecular features   

**Figure 2:**
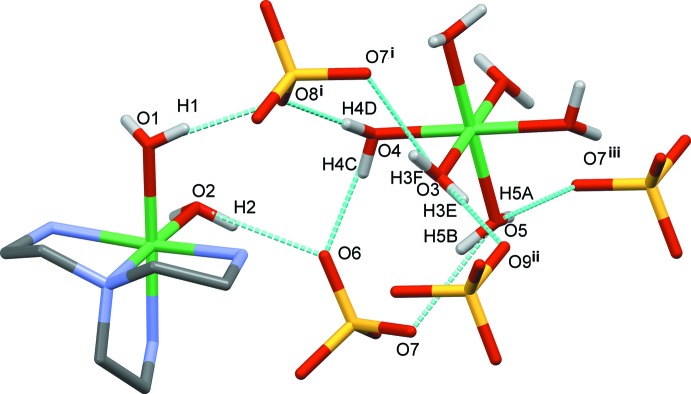
The hydrogen-bonding network (cyan dotted lines) in the title compound. Symmetry codes: (i) *x* + 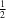, *y*, −*z* + 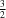; (ii) −*x* + 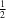, −*y* + 1, *z* − 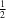; (iii) −*x* + 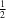, −*y* + 1, *z* + 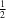.

The crystal structure of the title compound is consolidated through inter­molecular hydrogen bonding between the water mol­ecules from the [Ni(tren)(H_2_O)_2_] complex, the sulfate oxygen atoms and the water mol­ecules from the [Ni(H_2_O)_6_] complex (Fig. 2 ▸ and Table 1 ▸). In particular, the two water mol­ecules of [Ni(tren)(H_2_O)_2_] form O1—H1⋯O8^i^ and O2—H2⋯O6 hydrogen bonds of 2.05 (2) and 1.96 (2) Å respectively, involving two neighboring SO_4_^2−^ anions [symmetry code: (i) *x* + 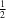, *y*, −*z* + 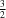). The [Ni(H_2_O)_6_] complex is hydrogen bonded to adjacent SO_4_^2−^ anions through O3—H3*E*⋯O9^ii^, O3—H3*F*⋯O7^i^, O4—H4*C*⋯O6, O4—H4*D*⋯O8^i^, O5—H5*B*⋯O7, O5—H5*A*⋯O7^iii^ contacts [symmetry codes: (ii) −*x* + 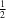, −*y* + 1, *z* − 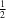; (iii) −*x* + 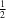, −*y* + 1, *z* + 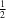]. These hydrogen-bond distances range from 1.905 (15) to 2.047 (18) Å. Additional weak hydrogen bonds are formed between the hydrogen atoms from the primary amine groups of the tren ligand and the sulfate oxygen atoms.

## Database survey   

A search for tris­(2-amino­eth­yl)aminenickel complexes in the Cambridge Structural Database (CSD version 5.38, updated February 2019; Groom *et al.*, 2016 ▸) yielded 222 hits. Among these results, 124 hits contained the ligand tris­(2-amino­eth­yl)amine capping the nickel ion, along with other types of ligands on the remaining coordination sites. Only two hits contain the di­aqua[tris­(2-amino­eth­yl)amine]nickel(II) complex, [Ni(tren)(H_2_O)_2_] (LUMVIY; Chen *et al.*, 2001 ▸; TIYQAT; Tanase *et al.*, 1996 ▸). More precisely, the asymmetric unit in LUMVIY comprises the [Ni(tren)(H_2_O)_2_]^2+^ cation with two independent halves of a 1,5-naphthalene­disulfonate (1,5nds) ligand as counter-anion. A common feature of this structure with the title compound is the hydrogen bond network formed between the water mol­ecules on the Ni(tren) motif with the counter anions. However, in the title compound, also the hydrogen atoms on the primary amine groups form hydrogen bonds with the sulfate anions, albeit quite weak. In TIYQAT, sulfate anions act as counter-ions for the [Ni(tren)(H_2_O)_2_]^2+^ complex, and uncoordinated water mol­ecules are included in the crystal lattice. The angle between the Ni center and the two oxygen atoms from the coordinated water mol­ecules are 86.52 (5)° (O7—Ni1—O8) and 86.9 (4)° (O5—Ni1—O6) for LUMVIY and TIYQAT, respectively. The corresponding angle O2—Ni—O1 in the tittle compound has a value of 88.70 (8)°, which is in good agreement with the reported values. The title compound is the first example of a crystal structure of [Ni(tren)(H_2_O)_2_]^2+^ co-crystallizing with the [Ni(H_2_O)_6_]^2+^ complex.

## Synthesis and crystallization   

**Figure 3:**
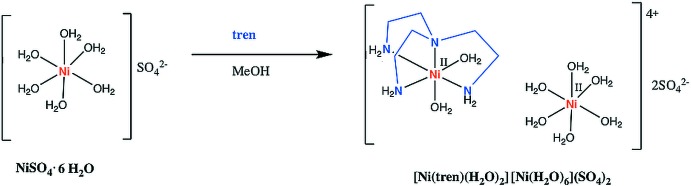
Reaction scheme for the synthesis of [Ni(tren)(H_2_O)_2_][Ni(H_2_O)_6_](SO_4_)_2._

**Figure 4:**
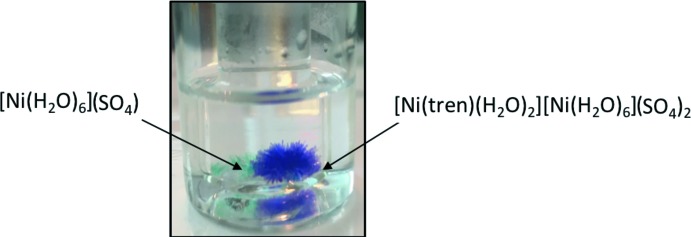
Crystallization of [Ni(tren)(H_2_O)_2_][Ni(H_2_O)_6_](SO_4_)_2_ and [Ni(H_2_O)_6_]SO_4_ in the same reaction vial.

The synthesis of the title compound is summarized in the reaction scheme shown in Fig. 3 ▸. NiSO_4_·6H_2_O and tris­(2-amino­eth­yl)amine (tren) were used without further purification. A methano­lic solution of NiSO_4_·6H_2_O (0.0265 g, 0.1 mmol) was added slowly to a tren (0.0146 g, 0.1 mmol) solution (4 mL MeOH) at room temperature. The resulting solution was stirred for two h and it changed color from light green to purple. The solution was then filtered through celite and evaporated under reduced pressure. Single crystals of the title compound were obtained by vapor diffusion of methanol into 2-propanol. In the crystallization process, two types of crystal were formed: the starting reagent hexa­hydrate nickel (II) complex (turquoise crystals) and the nickel(II) tren complex (purple crystals, Fig. 4 ▸). The reaction was performed both in a 1:1 and 1:2 metal–ligand molar ratio, always yielding the title compound. IR data: 3265 (*m*), 3171 (*m*), 2937 (*w*), 2891 (*w*), 1607 (*m*), 1472 (*w*) 1338 (*w*), 1054 (*s*), 984 (*m*), 885 (*m*), 750 (*w*), 685 (*w*).

## Refinement   

Crystal data, data collection and structure refinement details are summarized in Table 2 ▸. H atoms were included in geometrically calculated positions for the alkyl and amine groups using a riding model: C—H = 0.97 Å and N—H = 0.89 Å with *U*_iso_(H) =1.2*U*_eq_(C, N). The hydrogen atoms of the water mol­ecules were located from the difference-Fourier map; they were refined freely in the case of O1 and O2, with a DFIX of 0.85 (2) Å and *U*_iso_(H) =1.5*U*_eq_(O) in the case of O3 and O4, and riding with O—H = 0.88 Å and *U*_iso_(H) =1.5*U*_eq_(O) in the case of O5.

The N3 and C3 atoms of the tren ligand lie on a mirror plane perpendicular to [010]. This results in a symmetry-induced disorder of the N3/C4/C3 fragment.

## Supplementary Material

Crystal structure: contains datablock(s) I. DOI: 10.1107/S2056989020001358/xi2016sup1.cif

CCDC reference: 1911584

Additional supporting information:  crystallographic information; 3D view; checkCIF report

## Figures and Tables

**Table 1:** Hydrogen-bond geometry (Å, °)

*D*—H⋯*A*	*D*—H	H⋯*A*	*D*⋯*A*	*D*—H⋯*A*
O1—H1⋯O8^i^	0.78 (2)	2.05 (2)	2.8212 (16)	172 (2)
O2—H2⋯O6	0.81 (2)	1.96 (2)	2.7342 (15)	162 (3)
O3—H3*E*⋯O9^ii^	0.81 (2)	1.94 (2)	2.731 (2)	167 (2)
O3—H3*F*⋯O7^i^	0.85 (2)	2.05 (2)	2.8403 (18)	155 (2)
O4—H4*C*⋯O6	0.83 (2)	1.91 (2)	2.7249 (18)	171 (2)
O4—H4*D*⋯O8^i^	0.83 (2)	1.95 (2)	2.7810 (18)	179 (2)
O5—H5*A*⋯O7^iii^	0.88	2.02	2.8125 (19)	150
O5—H5*B*⋯O7	0.88	1.95	2.7826 (17)	160

Symmetry codes: (i) 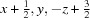; (ii) 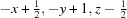; (iii) 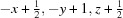.

**Table 2:** Experimental details

Crystal data	
Chemical formula	[Ni(C_6_H_18_N_4_)(H_2_O)_2_][Ni(H_2_O)_6_](SO_4_)_2_
*M*_r_	599.91
Crystal system, space group	Orthorhombic, *P* *n* *m* *a*
Temperature (K)	293
*a*, *b*, *c* (Å)	11.8937 (1), 21.3933 (2), 8.4468 (1)
*V* (Å^3^)	2149.25 (4)
*Z*	4
Radiation type	Cu *K*α
μ (mm^−1^)	4.76
Crystal size (mm)	0.28 × 0.21 × 0.09

Data collection	
Diffractometer	Rigaku Oxford Diffraction SuperNova, Single source at offset/far, HyPix3000
Absorption correction	Multi-scan (*CrysAlis PRO*; Rigaku OD, 2015 ▸)
*T*_min_, *T*_max_	0.353, 0.661
No. of measured, independent and observed [*I* > 2σ(*I*)] reflections	17858, 2044, 1996
*R*_int_	0.023
(sin θ/λ)_max_ (Å^−1^)	0.605

Refinement	
*R*[*F* ^2^ > 2σ(*F* ^2^)], *wR*(*F* ^2^), *S*	0.023, 0.063, 1.12
No. of reflections	2044
No. of parameters	173
No. of restraints	8
H-atom treatment	All H-atom parameters refined
Δρ_max_, Δρ_min_ (e Å^−3^)	0.37, −0.35

Computer programs: *CrysAlis PRO* (Rigaku OD, 2015 ▸), *olex2.solve* (Bourhis *et al.*, 2015 ▸), *SHELXL2016* (Sheldrick, 2015 ▸) and *OLEX2* (Dolomanov *et al.*, 2009 ▸).

## supplementary crystallographic information

### Crystal data

**Table:**

[Ni(C_6_H_18_N_4_)(H_2_O)_2_][Ni(H_2_O)_6_](SO_4_)_2_	*D*_x_ = 1.854 Mg m^−^^3^
*M**_r_* = 599.91	Cu *K*α radiation, λ = 1.54184 Å
Orthorhombic, *P**n**m**a*	Cell parameters from 14387 reflections
*a* = 11.8937 (1) Å	θ = 3.7–68.8°
*b* = 21.3933 (2) Å	µ = 4.76 mm^−^^1^
*c* = 8.4468 (1) Å	*T* = 293 K
*V* = 2149.25 (4) Å^3^	Block, clear violet
*Z* = 4	0.28 × 0.21 × 0.09 mm
*F*(000) = 1256	

### Data collection

**Table:**

Rigaku Oxford Diffraction SuperNova, Single source at offset/far, HyPix3000 diffractometer	1996 reflections with *I* > 2σ(*I*)
ω scans	*R*_int_ = 0.023
Absorption correction: multi-scan (CrysAlis PRO; Rigaku OD, 2015)	θ_max_ = 68.9°, θ_min_ = 4.1°
*T*_min_ = 0.353, *T*_max_ = 0.661	*h* = −14→14
17858 measured reflections	*k* = −25→25
2044 independent reflections	*l* = −10→10

### Refinement

**Table:**

Refinement on *F*^2^	Secondary atom site location: difference Fourier map
Least-squares matrix: full	Hydrogen site location: mixed
*R*[*F*^2^ > 2σ(*F*^2^)] = 0.023	All H-atom parameters refined
*wR*(*F*^2^) = 0.063	*w* = 1/[σ^2^(*F*_o_^2^) + (0.0312*P*)^2^ + 1.2986*P*] where *P* = (*F*_o_^2^ + 2*F*_c_^2^)/3
*S* = 1.12	(Δ/σ)_max_ < 0.001
2044 reflections	Δρ_max_ = 0.37 e Å^−^^3^
173 parameters	Δρ_min_ = −0.35 e Å^−^^3^
8 restraints	Extinction correction: SHELXL2016 (Sheldrick, 2015), Fc^*^=kFc[1+0.001xFc^2^λ^3^/sin(2θ)]^-1/4^
Primary atom site location: dual	Extinction coefficient: 0.00044 (5)

### Special details

**Table:**

Geometry. All esds (except the esd in the dihedral angle between two l.s. planes) are estimated using the full covariance matrix. The cell esds are taken into account individually in the estimation of esds in distances, angles and torsion angles; correlations between esds in cell parameters are only used when they are defined by crystal symmetry. An approximate (isotropic) treatment of cell esds is used for estimating esds involving l.s. planes.

### Fractional atomic coordinates and isotropic or equivalent isotropic displacement parameters (Å^2^)

**Table:**

	*x*	*y*	*z*	*U*_iso_*/*U*_eq_	Occ. (<1)
Ni1	0.31517 (3)	0.250000	0.58049 (4)	0.01987 (12)	
O1	0.49430 (15)	0.250000	0.5573 (2)	0.0285 (4)	
H1	0.523 (2)	0.2795 (11)	0.592 (3)	0.042 (7)*	
O2	0.33527 (17)	0.250000	0.8268 (2)	0.0315 (4)	
H2	0.304 (2)	0.2795 (11)	0.867 (3)	0.056 (8)*	
N1	0.29062 (16)	0.250000	0.3386 (2)	0.0237 (4)	
N2	0.32729 (13)	0.34830 (7)	0.55183 (18)	0.0291 (3)	
H2A	0.275056	0.367313	0.609911	0.035*	
H2B	0.394537	0.361563	0.583583	0.035*	
N3	0.14137 (18)	0.250000	0.5913 (3)	0.0330 (5)	
H3A	0.116429	0.211740	0.613150	0.040*	0.5
H3B	0.118127	0.275722	0.667351	0.040*	0.5
C1	0.34686 (17)	0.30780 (9)	0.2826 (2)	0.0336 (4)	
H1A	0.327506	0.315269	0.172689	0.040*	
H1B	0.427761	0.302752	0.289509	0.040*	
C2	0.31074 (17)	0.36314 (9)	0.3821 (2)	0.0362 (4)	
H2C	0.354856	0.399608	0.353854	0.043*	
H2D	0.232177	0.372476	0.362353	0.043*	
C3	0.1684 (2)	0.250000	0.3008 (3)	0.0344 (6)	
H3C	0.145952	0.208172	0.269741	0.041*	0.5
H3D	0.154994	0.277594	0.211689	0.041*	0.5
C4	0.0975 (3)	0.27067 (19)	0.4375 (5)	0.0384 (10)	0.5
H4A	0.092804	0.315934	0.437065	0.046*	0.5
H4B	0.022018	0.254369	0.424014	0.046*	0.5
Ni2	0.500000	0.500000	1.000000	0.02058 (12)	
O3	0.46413 (12)	0.51163 (6)	0.76229 (15)	0.0337 (3)	
H3E	0.4398 (19)	0.5433 (9)	0.723 (2)	0.051*	
H3F	0.5103 (18)	0.4984 (11)	0.693 (2)	0.051*	
O4	0.47512 (11)	0.40611 (6)	0.96546 (16)	0.0291 (3)	
H4C	0.4079 (14)	0.3972 (9)	0.956 (3)	0.044*	
H4D	0.5051 (17)	0.3935 (9)	0.883 (2)	0.044*	
O5	0.33269 (10)	0.51618 (6)	1.05567 (15)	0.0309 (3)	
H5A	0.320545	0.510448	1.156884	0.046*	
H5B	0.288625	0.490680	1.003402	0.046*	
S1	0.14894 (3)	0.39419 (2)	0.92733 (4)	0.02016 (12)	
O6	0.26060 (10)	0.36508 (6)	0.92041 (16)	0.0339 (3)	
O7	0.15935 (10)	0.46130 (5)	0.88353 (15)	0.0297 (3)	
O8	0.07525 (11)	0.36247 (6)	0.81077 (16)	0.0341 (3)	
O9	0.10124 (13)	0.38801 (6)	1.08500 (15)	0.0408 (4)	

### Atomic displacement parameters (Å^2^)

**Table:**

	*U*^11^	*U*^22^	*U*^33^	*U*^12^	*U*^13^	*U*^23^
Ni1	0.0238 (2)	0.0199 (2)	0.0160 (2)	0.000	−0.00128 (15)	0.000
O1	0.0262 (9)	0.0235 (9)	0.0357 (10)	0.000	−0.0058 (8)	0.000
O2	0.0465 (11)	0.0277 (10)	0.0203 (9)	0.000	−0.0002 (8)	0.000
N1	0.0257 (10)	0.0280 (10)	0.0172 (9)	0.000	−0.0027 (8)	0.000
N2	0.0326 (8)	0.0234 (7)	0.0313 (8)	0.0027 (6)	−0.0018 (6)	−0.0024 (6)
N3	0.0275 (11)	0.0381 (12)	0.0335 (12)	0.000	0.0076 (9)	0.000
C1	0.0396 (10)	0.0397 (11)	0.0214 (9)	−0.0057 (8)	0.0016 (8)	0.0098 (8)
C2	0.0439 (10)	0.0254 (9)	0.0392 (11)	−0.0015 (8)	−0.0059 (9)	0.0115 (8)
C3	0.0316 (13)	0.0423 (15)	0.0292 (13)	0.000	−0.0114 (11)	0.000
C4	0.0252 (16)	0.046 (2)	0.044 (2)	0.0077 (14)	−0.0074 (15)	−0.0045 (16)
Ni2	0.0226 (2)	0.0206 (2)	0.0185 (2)	−0.00029 (15)	−0.00085 (15)	−0.00050 (15)
O3	0.0442 (8)	0.0364 (7)	0.0206 (6)	0.0102 (6)	−0.0007 (6)	0.0031 (5)
O4	0.0297 (6)	0.0264 (6)	0.0312 (7)	−0.0037 (5)	0.0030 (6)	−0.0036 (5)
O5	0.0258 (6)	0.0392 (7)	0.0279 (6)	−0.0029 (5)	0.0000 (5)	−0.0073 (6)
S1	0.0224 (2)	0.0187 (2)	0.0193 (2)	−0.00018 (14)	−0.00065 (14)	0.00073 (14)
O6	0.0255 (6)	0.0294 (7)	0.0468 (8)	0.0032 (5)	−0.0021 (6)	−0.0059 (6)
O7	0.0369 (7)	0.0207 (6)	0.0316 (7)	−0.0039 (5)	−0.0083 (5)	0.0049 (5)
O8	0.0378 (7)	0.0280 (6)	0.0364 (7)	−0.0079 (5)	−0.0130 (6)	0.0017 (5)
O9	0.0594 (9)	0.0344 (7)	0.0284 (7)	0.0040 (7)	0.0168 (6)	0.0024 (6)

### Geometric parameters (Å, º)

**Table:**

Ni1—O1	2.1395 (18)	C2—H2D	0.9700
Ni1—O2	2.0940 (19)	C3—H3C	0.9700
Ni1—N1	2.0640 (19)	C3—H3C^i^	0.9700
Ni1—N2	2.1217 (15)	C3—H3D	0.9700
Ni1—N2^i^	2.1217 (15)	C3—H3D^i^	0.9700
Ni1—N3	2.069 (2)	C3—C4	1.496 (4)
O1—H1	0.78 (2)	C4—H4A	0.9700
O1—H1^i^	0.78 (2)	C4—H4B	0.9700
O2—H2	0.81 (2)	Ni2—O3^ii^	2.0678 (13)
O2—H2^i^	0.81 (2)	Ni2—O3	2.0678 (13)
N1—C1	1.483 (2)	Ni2—O4^ii^	2.0511 (13)
N1—C1^i^	1.483 (2)	Ni2—O4	2.0511 (13)
N1—C3	1.488 (3)	Ni2—O5	2.0739 (12)
N2—H2A	0.8900	Ni2—O5^ii^	2.0739 (12)
N2—H2B	0.8900	O3—H3E	0.808 (15)
N2—C2	1.481 (2)	O3—H3F	0.851 (15)
N3—H3A^i^	0.8900	O4—H4C	0.826 (15)
N3—H3A	0.8900	O4—H4D	0.830 (15)
N3—H3B	0.8900	O5—H5A	0.8756
N3—H3B^i^	0.8900	O5—H5B	0.8759
N3—C4	1.468 (4)	S1—O6	1.4679 (13)
C1—H1A	0.9700	S1—O7	1.4878 (12)
C1—H1B	0.9700	S1—O8	1.4826 (12)
C1—C2	1.514 (3)	S1—O9	1.4537 (13)
C2—H2C	0.9700		
			
O2—Ni1—O1	88.70 (8)	C1—C2—H2D	109.8
O2—Ni1—N2	96.06 (4)	H2C—C2—H2D	108.2
O2—Ni1—N2^i^	96.06 (4)	N1—C3—H3C^i^	109.06 (3)
N1—Ni1—O1	92.87 (8)	N1—C3—H3C	109.1
N1—Ni1—O2	178.42 (8)	N1—C3—H3D^i^	109.07 (10)
N1—Ni1—N2^i^	84.07 (4)	N1—C3—H3D	109.1
N1—Ni1—N2	84.07 (4)	N1—C3—C4	112.6 (2)
N1—Ni1—N3	84.39 (9)	H3C—C3—H3C^i^	134.6
N2^i^—Ni1—O1	85.52 (4)	H3C^i^—C3—H3D^i^	107.8
N2—Ni1—O1	85.52 (4)	H3C—C3—H3D	107.8
N2^i^—Ni1—N2	164.74 (9)	H3C—C3—H3D^i^	35.2
N3—Ni1—O1	177.27 (8)	H3D—C3—H3C^i^	35.2
N3—Ni1—O2	94.03 (9)	H3D—C3—H3D^i^	75.0
N3—Ni1—N2^i^	94.18 (4)	C4—C3—H3C	109.1
N3—Ni1—N2	94.18 (4)	C4—C3—H3C^i^	77.37 (16)
Ni1—O1—H1^i^	113.6 (18)	C4—C3—H3D^i^	133.34 (17)
Ni1—O1—H1	113.6 (18)	C4—C3—H3D	109.1
H1—O1—H1^i^	109 (3)	N3—C4—H3A^i^	34.21 (10)
Ni1—O2—H2	111.3 (19)	N3—C4—C3	113.2 (3)
Ni1—O2—H2^i^	111.3 (19)	N3—C4—H4A	108.9
H2—O2—H2^i^	103 (4)	N3—C4—H4B	108.9
C1^i^—N1—Ni1	104.58 (11)	C3—C4—H3A^i^	136.9 (3)
C1—N1—Ni1	104.58 (11)	C3—C4—H4A	108.9
C1—N1—C1^i^	113.0 (2)	C3—C4—H4B	108.9
C1—N1—C3	111.85 (12)	H4A—C4—H3A^i^	76.7
C1^i^—N1—C3	111.85 (12)	H4A—C4—H4B	107.8
C3—N1—Ni1	110.50 (15)	H4B—C4—H3A^i^	109.6
Ni1—N2—H2A	110.0	O3^ii^—Ni2—O3	180.0
Ni1—N2—H2B	110.0	O3^ii^—Ni2—O5	89.88 (5)
H2A—N2—H2B	108.4	O3—Ni2—O5	90.12 (5)
C2—N2—Ni1	108.29 (11)	O3^ii^—Ni2—O5^ii^	90.12 (5)
C2—N2—H2A	110.0	O3—Ni2—O5^ii^	89.88 (5)
C2—N2—H2B	110.0	O4—Ni2—O3^ii^	92.87 (5)
Ni1—N3—H3A	110.0	O4^ii^—Ni2—O3	92.87 (5)
Ni1—N3—H3A^i^	110.008 (12)	O4—Ni2—O3	87.13 (5)
Ni1—N3—H3B^i^	110.01 (5)	O4^ii^—Ni2—O3^ii^	87.13 (5)
Ni1—N3—H3B	110.0	O4^ii^—Ni2—O4	180.0
H3A—N3—H3A^i^	133.8	O4^ii^—Ni2—O5^ii^	93.28 (5)
H3A—N3—H3B	108.4	O4^ii^—Ni2—O5	86.72 (5)
H3A—N3—H3B^i^	34.7	O4—Ni2—O5^ii^	86.72 (5)
H3A^i^—N3—H3B^i^	108.4	O4—Ni2—O5	93.28 (5)
H3B—N3—H3A^i^	34.7	O5—Ni2—O5^ii^	180.00 (7)
H3B—N3—H3B^i^	76.4	Ni2—O3—H3E	124.9 (15)
C4—N3—Ni1	108.42 (19)	Ni2—O3—H3F	119.7 (15)
C4—N3—H3A	110.0	H3E—O3—H3F	103 (2)
C4—N3—H3A^i^	77.77 (16)	Ni2—O4—H4C	112.3 (14)
C4—N3—H3B	110.0	Ni2—O4—H4D	112.1 (14)
C4—N3—H3B^i^	135.62 (17)	H4C—O4—H4D	105 (2)
N1—C1—H1A	109.6	Ni2—O5—H5A	110.9
N1—C1—H1B	109.6	Ni2—O5—H5B	110.8
N1—C1—C2	110.31 (15)	H5A—O5—H5B	107.8
H1A—C1—H1B	108.1	O6—S1—O7	108.92 (8)
C2—C1—H1A	109.6	O6—S1—O8	108.32 (8)
C2—C1—H1B	109.6	O8—S1—O7	109.01 (7)
N2—C2—C1	109.38 (14)	O9—S1—O6	110.55 (9)
N2—C2—H2C	109.8	O9—S1—O7	110.37 (8)
N2—C2—H2D	109.8	O9—S1—O8	109.63 (8)
C1—C2—H2C	109.8		
			
Ni1—N1—C1—C2	−48.90 (17)	N1—C3—C4—N3	−35.8 (3)
Ni1—N1—C3—C4	18.68 (18)	C1^i^—N1—C1—C2	−162.01 (12)
Ni1—N2—C2—C1	−27.22 (18)	C1—N1—C3—C4	−97.4 (2)
Ni1—N3—C4—C3	34.2 (3)	C1^i^—N1—C3—C4	134.7 (2)
N1—C1—C2—N2	52.2 (2)	C3—N1—C1—C2	70.7 (2)

Symmetry codes: (i) *x*, −*y*+1/2, *z*; (ii) −*x*+1, −*y*+1, −*z*+2.

### Hydrogen-bond geometry (Å, º)

**Table:**

*D*—H···*A*	*D*—H	H···*A*	*D*···*A*	*D*—H···*A*
O1—H1···O8^iii^	0.78 (2)	2.05 (2)	2.8212 (16)	172 (2)
O2—H2···O6	0.81 (2)	1.96 (2)	2.7342 (15)	162 (3)
O3—H3*E*···O9^iv^	0.81 (2)	1.94 (2)	2.731 (2)	167 (2)
O3—H3*F*···O7^iii^	0.85 (2)	2.05 (2)	2.8403 (18)	155 (2)
O4—H4*C*···O6	0.83 (2)	1.91 (2)	2.7249 (18)	171 (2)
O4—H4*D*···O8^iii^	0.83 (2)	1.95 (2)	2.7810 (18)	179 (2)
O5—H5*A*···O7^v^	0.88	2.02	2.8125 (19)	150
O5—H5*B*···O7	0.88	1.95	2.7826 (17)	160

Symmetry codes: (iii) *x*+1/2, *y*, −*z*+3/2; (iv) −*x*+1/2, −*y*+1, *z*−1/2; (v) −*x*+1/2, −*y*+1, *z*+1/2.
